# Distribution and predictors of wing shape and size variability in three sister species of solitary bees

**DOI:** 10.1371/journal.pone.0173109

**Published:** 2017-03-08

**Authors:** Simon Dellicour, Maxence Gerard, Jérôme G. Prunier, Alexandre Dewulf, Michael Kuhlmann, Denis Michez

**Affiliations:** 1 Rega Institute for Medical Research, Clinical and Epidemiological Virology, Department of Microbiology and Immunology, KU Leuven—University of Leuven, Minderbroedersstaat 10, Leuven, Belgium; 2 *Laboratoire de Zoologie*, Research institute of Biosciences, University of Mons, Place du Parc 23, Mons, Belgium; 3 Station d'Écologie Théorique et Expérimentale, Université de Toulouse, CNRS, Moulis, France; 4 Zoological Museum, University of Kiel, Hegewischstr. 3, Kiel, Germany; 5 Department of Life Sciences, Natural History Museum, Cromwell Road, London, United Kingdom; University of Innsbruck, AUSTRIA

## Abstract

Morphological traits can be highly variable over time in a particular geographical area. Different selective pressures shape those traits, which is crucial in evolutionary biology. Among these traits, insect wing morphometry has already been widely used to describe phenotypic variability at the inter-specific level. On the contrary, fewer studies have focused on intra-specific wing morphometric variability. Yet, such investigations are relevant to study potential convergences of variation that could highlight micro-evolutionary processes. The recent sampling and sequencing of three solitary bees of the genus *Melitta* across their entire species range provides an excellent opportunity to jointly analyse genetic and morphometric variability. In the present study, we first aim to analyse the spatial distribution of the wing shape and centroid size (used as a proxy for body size) variability. Secondly, we aim to test different potential predictors of this variability at both the intra- and inter-population levels, which includes genetic variability, but also geographic locations and distances, elevation, annual mean temperature and precipitation. The comparison of spatial distribution of intra-population morphometric diversity does not reveal any convergent pattern between species, thus undermining the assumption of a potential local and selective adaptation at the population level. Regarding intra-specific wing shape differentiation, our results reveal that some tested predictors, such as geographic and genetic distances, are associated with a significant correlation for some species. However, none of these predictors are systematically identified for the three species as an important factor that could explain the intra-specific morphometric variability. As a conclusion, for the three solitary bee species and at the scale of this study, our results clearly tend to discard the assumption of the existence of a common pattern of intra-specific signal/structure within the intra-specific wing shape and body size variability.

## Introduction

Variability in morphological traits has been considered as a cornerstone in evolutionary biology. Their variability in shape and size has been widely studied to characterise phenotypic diversity (e.g. [1]), evolution of an organism (e.g. [2]) or taxon delimitation (e.g. [3]). Different traits have been investigated in their shape and size variability but insect wings have been selected in many studies, especially in systematics (e.g. [4]) and palaeontology (e.g. [5]). Insect wings are particularly easy to study because they are flat, two-dimensional structures with many homologous landmarks (e.g. cross veins). In addition, insects constitute the most diversified animal group and they present a particularly high degree of variability in their wing venation at many taxonomic levels ranging from order (e.g. Hymenoptera) to populations (e.g. [6]).

Wing shape and size are functionally essential traits for flight performance [7, 8], foraging and dispersal abilities [9, 10]. As a consequence, these traits are under adaptive selection pressure [11] though sexual selection may also be influential in some species [12, 13]. Indeed, in those microevolutionary processes, genetic variability and environmental factors are thought to potentially impact morphological traits [14]. Intra-specific shape variations can be observed when local conditions may select particular shapes, e.g., shapes associated with fragmented habitats [14], temperature [15], precipitation [16] or elevation [17]. Intra-specific shape variation may also be related to internal factors like genetic diversity [18] or body size (i.e. allometry [19]), and developmental factors such as temperature [20]. Regarding the wing size, it can also be influenced by environmental and internal factors at the inter- and intra-specific levels [21]. While many studies have focused on wing shape and size variability at the inter-specific level (e.g. species diagnose [4]), only a few studies have investigated the variability of these two related traits simultaneously at the intra-specific level in different sister species [22, 23]. This approach is crucial to detect potential convergence of variation that could highlight micro-evolutionary processes.

In the context of a recent comparative phylogeographic study [24], three solitary and sister species of the genus *Melitta* have been sampled on their entire range and sequenced at one mitochondrial and four nuclear genes. This dataset represents an interesting opportunity to perform a study of intra-specific morphometric variability. More specifically, the overall goal of the present study is to use a geometric morphometric approach, in order to explore the intra-specific wing shape and size variability in the sister species *Melitta leporina*, *M*. *nigricans* and *M*. *tricincta* [24]. The study of the intra-specific wing shape variability across the species range of three solitary bees will allow assessing the presence of spatial structures for this morphometric variability, but also to test whether it can be explained by biotic and abiotic factors.

Firstly, we aim to investigate and compare among species the spatial pattern of wing shape and size variability at the intra-specific level. In this study, we defined (morphometric/genetic) *variability* as the combination of two distinct aspects: intra-population *diversity* and inter-population *differentiation* (or *distance*). In particular, we aim to analyse the spatial distribution of inter-population wing shape differentiation and intra-population wing shape diversity. Under the assumption of environmental pressures acting on wing morphometry, common patterns of spatial distribution of wing shape variability should be observed among the three species.

Secondly, we aim to assess the influence of different potential predictors (geographic and genetic distance, differences in elevation, mean annual temperature and precipitation) on the intra-specific wing shape variability. The choice of predictors to test is based on several hypotheses about biotic and abiotic factors likely to influence variability in wing shape, at both intra- and inter-population levels. More specifically, we aim to test (i) if wing shape differentiation is correlated to geographic distances [25], (ii) if wing shape diversity/differentiation is correlated to genetic diversity/differentiation [26], and (iii) if important environmental factors as elevation, temperature and elevation could explain the intra-specific wing shape variability [16, 17]. Regarding the latter question, our hypothesis is that different selective pressures across the species range should lead to different wing shape reaction norms corresponding to different eco-morphological adaptive optima.

Finally, we also aim to test the same potential predictors on the intra-specific wing size variability. In particular, we are interested in testing the correlation between wing size and mean annual temperature. Indeed, in the case of insects, wing size can be used as a proxy for body size [13] and thus allows to test if body size and temperature are negatively correlated as described in Bergmann’s rule [27] for other organisms. Originally formulated for endotherm vertebrates, this rule describes “an ecogeographical pattern where organisms show increased body size or mass in colder climates, reflected in a latitudinal cline with larger organisms at higher latitudes” [28]. Despite a relatively clear consensus for endotherms, the relationship defined by Bergmann’s rule is less clear for ectotherms as insects [29]. Yet, since several bee species are for instance able of thorax thermoregulation to maintain flight performances [30–31], insects cannot be universally considered as ectotherms. Whether we consider ecto- or endothermy for insects, previous studies at the intra-specific level already confirmed (e.g. [32]) or rejected (e.g. [33]) this rule for insect species. Although Bergmann’s rule was initially formulated for endotherm vertebrates, we hereafter use the “Bergmann cline” expression to designate a negative relation between body size and temperature.

## Materials and methods

### Selected biological models, sampling and DNA sequence datasets

*Melitta leporina*, *M*. *nigricans* and *M*. *tricincta* are three sister species [34] considered as floral specialists [35]. *M*. *leporina* is specialised on the flowers of Fabaceae, and *M*. *nigricans* and *M*. *tricincta* on *Lythrum* (Lythraceae) and *Odontites* (Orobanchaceae), respectively [35]. They are very similar in all other ecological traits (e.g. nesting behaviour). However, they display different levels of genetic variability: *M*. *leporina* exhibits a higher level of genetic diversity potentially related to a higher effective population size allowed by its more abundant food resource [24].

**Fig 1:**
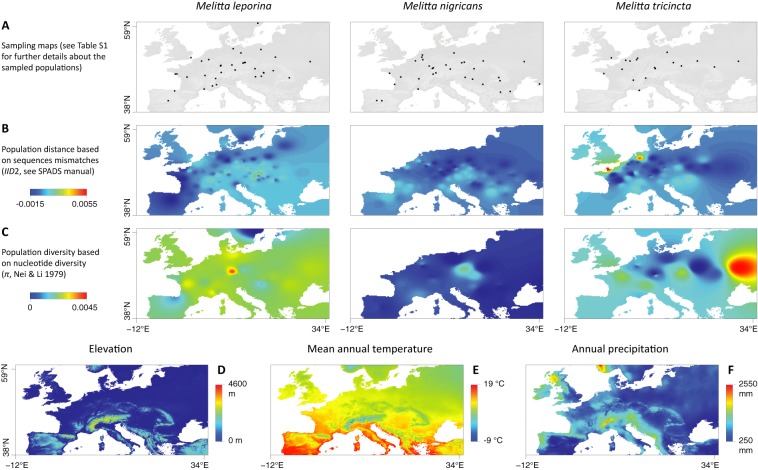
**Sampling map for each of the three Melitta species (A), inter-population genetic distance based on DNA sequence mismatches (*IID*2; B) and population nucleotide diversity (*π*; C) interpolation graphs generated with a distance weighting parameter *a* = 5, as well as maps displaying the elevation (D), mean annual temperature (E) and annual precipitation (F) on the study area.** Graphs based on inter-population distances were generated with the GDisPAL function and those based on population diversity with the GDivPAL function (see the text for further details). See also Fig A-B in S1 File for the graphs generated with *a* = 1 and 10.

Male specimens of these three species were sampled across their entire species range for the purpose of a previous comparative phylogeographic study [24]. No specific permissions were required for the different sampling locations and none of the three species is considered endangered. For this previous study, sampled individuals were sequenced at one mitochondrial and four nuclear genes (see [24] for the detailed procedure as well as for detailed analyses and discussion about the genetic variability of these five gene fragments). In the present study, we focused on the West-Palearctic range of these three species, which also corresponds to their co-distribution area (i.e. excluding the East-Palearctic populations sampled for *M*. *leporina*; see Fig 1A). In addition, we excluded individuals for which we were not able to obtain morphometric data (see below). The final dataset included 104, 118 and 64 haploid male individuals sampled for *M*. *leporina*, *nigricans* and *tricincta*, respectively (see Table A in S1 File for detailed information about the sampled localities).

### Measuring wing shape morphometric and body size variability

**Fig 2:**
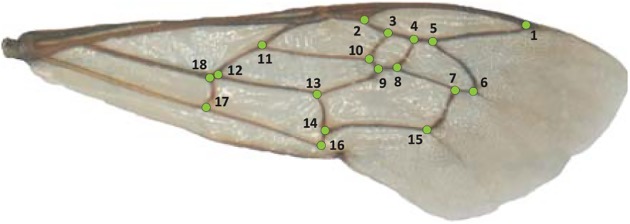
Right forewing of a *Melitta leporina* male specimen with the 18 landmarks selected to describe the shape.

Pictures of the right and left forewings of each specimen were taken using a D70 Nikon (Shinjuku, Japan) coupled with an Olympus SZ010 binocular with an AF-S NIKKOR 18–105 millimetres (Shinjuku, Japan) and GWH10X-CD oculars. We used a magnification of 0.7 and an illumination of 3 out of 5. Photographs were uploaded to tps-UTIL 1.46. Wing shapes were digitised with two-dimensional Cartesian coordinates of 18 landmarks on wing veins (Fig 2) using tps-DIG 2.1 [36, 37]. We applied the generalized least square procrustes superimposition method to remove all non-shape differences (i.e. scale, translation and rotation of the 286 landmark configurations against the consensus configuration [38]). The aligned landmark configurations were projected into the Euclidean space tangent to the curved Kendall’s shape space for further statistical analyses. The correlation coefficient between the procrustes distances (i.e. the square root of the sum of squared distances between pairs of corresponding landmarks) in the shape space and the Euclidean distances in the linear tangent space equalled 1. This indicated that the curvature of the shape space around our data was negligible [39]. The least-squares regression slope through the origin and the correlation coefficient between the two distances were calculated with tps-SMALL v1.25 [40].

Morphometric analyses were based on only one of the two wings per specimen because two measurements on a same individual cannot be considered as independent. We always used the right wing when it was available; in the case where the right wing was damaged, we used the left wing. Procrustes distances estimated in the linear tangent space were used to summarise the shape differences between two landmark configurations and to build *inter-individual* morphometric distance matrices. These morphometric and statistical methods were performed using functions of the R package “geomorph” [41].

As a first preliminary analysis, we explored the morphometric differentiation at the inter-specific level by analysing all the inter-individual distances computed between all the individuals sampled for the three species with a principal coordinates analysis (PCoA). After this first analysis, inter-individual morphometric distances *d* were only considered at the intra-specific level. Intra-specific wing shape variability was investigated here by estimating the inter-population morphometric distances and intra-population morphometric diversity, two metrics defined below.

*Inter-population* morphometric distance *mD* between two populations *j*_*1*_ and *j*_*2*_ was computed by summing the distances between each pair of individuals belonging to a different population and dividing this value by the number of pairwise distances considered:

$${mD}_{j_{1}j_{2}}=\frac{1}{n_{j_{1}}n_{j_{2}}}\sum_{i_{1}=1}^{n_{j_{1}}}\sum_{i_{2}=1}^{n_{j_{2}}}d_{i_{1}i_{2}}$$

where *n*_*j1*_ and *n*_*j2*_ are the numbers of individuals sampled in populations *j*_*1*_ and *j*_*2*_, and *d*_*i1i2*_ the inter-individual morphometric distance between individuals *i*_*1*_ and *i*_*2*_, respectively sampled in populations *j*_*1*_ and *j*_*2*_. Similarly, morphometric diversity *md* within a population *j* was computed as the mean of pairwise distances between two individuals sampled in this population as followed:

$${md}_{j}=\frac{2}{n_{j}(n_{j}-1)}\sum_{i_{1}\neq i_{2}}d_{i_{1}i_{2}}$$

where *n*_*j*_ is the number of individuals sampled in populations *j*, and *d*_*i1i2*_ the inter-individual morphometric distance between two different individuals *i*_*1*_ and *i*_*2*_ both sampled in population *j*. It is worth noting that these diversity and distance indices are independent from sample size, which varies among species and populations.

We used the wing centroid size as a proxy for body size [13]. Wing centroid size is defined as the square root of the sum of squared distances between all landmarks and their centroid [42]. We estimated the centroid size for each selected wing (one per individual; see above) and, in order to perform the same analyses as for the morphometric distances based on wing shape morphometry (*mD*’s and *md*’s), we computed the mean wing size difference *wsD* between each pair of populations as well as the mean wing size *wsM* for each population.

### Mapping and comparing the morphometric variability across species ranges

To compare morphometric wing shape and body size variability across the range of the three species, we first generated interpolation graphs to perform preliminary visual comparisons. These interpolation graphs aim to display the geographic distribution of inter-population morphometric distances as well as intra-population morphometric diversity. Interpolation of wing shape diversity was based on *md* values estimated within each sampled population, while for wing shape differentiation, interpolation was based on *mD* values assigned at the midpoint of each edge of a connectivity network (i.e. a Delaunay triangulation) applied to sampled populations. In addition, we also used this interpolation procedure to map the spatial evolution of wing size variability (*wsM*). All interpolations were generated on a the same template raster (resolution: 10 arcmin) using three values for distance weighting parameter *a* (1, 5 and 10) and all interpolations were based on great circle geographic distances (i.e. distances on the surface of the Earth) measured in kilometres and estimated using the R package “fields” [43]. Furthermore, as advised by [44], we performed the inter-population morphometric distance interpolations using residual distances derived from the linear regression of genetic vs. geographical distances [45]. All the interpolation graphs were generated using an extension of a method developed by [46], based on an interpolation procedure (inverse distance-weighted interpolation; [47, 48]), and implemented in the R functions GDisPAL and GDivPAL available with the toolbox SPADS 1.0 [49].

In order to provide similar preliminary visual comparisons between morphometric and genetic variability, we also reported maps displaying the geographic distribution of inter-population genetic distances and intra-population genetic diversity based on the same sampling scheme (Fig 1B and 1C). Similar maps were already presented in [24]. However, the versions presented here are only based on individuals for which we also obtained morphometric data. These surfaces were based on genetic distances and diversity indices estimated with SPADS [49]: the inter-individual distances *IID*2 (based on DNA sequence mismatches and averaged over the different loci; see SPADS manual for further details) and the nucleotide diversity *π* [50], respectively.

**Fig 3:**
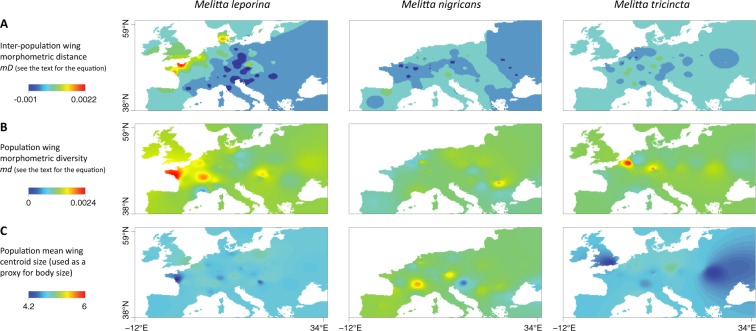
**Interpolation graphs generated with a distance weighting parameter *a* = 5:** inter-population morphometric distance (*mD*; **A**), population morphometric diversity (*md*; **B**) and population mean wing centroid size (*wsM*, used as a proxy for body size; **C**). Graphs based on inter-population distances were generated with the GDisPAL function and those based on population diversity with the GDivPAL function (see the text for further details). See also Figs A and B in S1 File for equivalent interpolation graphs generated with *a* = 1 and 10.

In addition to the preliminary visual comparison between interpolation maps generated for each species, we also estimated, for each pair of species, correlation statistics between intra-population morphometric diversity values reported at the same locations. As these species were not sampled at the exact same locations, these correlation statistics were based on values extracted from interpolation graphs at the same locations. To avoid as much extrapolation as possible, we only considered morphometric diversity values reported for sampling localities. In practice, *md* values were extracted from the corresponding interpolation maps (Fig 3B, maps generated with a distance weighting parameter *a* = 5) with the combined set of sampling geographic coordinates of all the three species (total of 83 localities). For this analysis, we estimated Pearson’s correlation coefficients as well as linear regression determination coefficients and their associated p-value.

### Testing potential predictors of wing shape and size variability

We tested the correlation between some specific sets of potential predictors and four distinct response variables: (i) inter-population morphometric distances *mD*’s, (ii) intra-population morphometric diversity *md*’s, (iii) inter-population mean wing size differences *wsD*’s and (iv) population mean wing size *wsD*’s. For the inter-population response variables (*mD* and *wsD*), we tested the following potential predictors: inter-population genetic distances based on DNA sequence mismatches (*IID*2 that is, a proxy for genetic differentiation), great circle geographic distances (predictor for isolation-by-distance, IBD [51]), as well as pairwise differences in elevation, mean annual temperature and annual precipitation (predictors for isolation-by-environment, IBE; e.g. [52]; see also Table 1). For the intra-population response variables (*md* and *wsM*), we tested the following potential predictors: longitude, latitude, nucleotide diversity *π* [50], elevation, annual mean temperature and annual precipitation (Table 1). Annual mean temperature and annual precipitation are two bioclimatic variables extracted from the WorldClim database (WorldClim 1.4 [53]; see Fig 1D and 1F for the mapping of the three later variables on the study area). In any case, wing size variability and wing shape variability were also considered as possible predictors of each other. We performed multiple regressions on distance matrices (MRDM) for inter-population variables and linear regression (LR) for intra-population variables, both coupled with commonality analyses (CA [54]), hereafter respectively referenced as “MRDM-CA” and “LR-CA” [55]. Note that all these analyses were also performed by replacing the “annual mean temperature” data by the mean temperature of the warmest quarter (bioclimatic variable “bio10” of the WorldClim database). These additional analyses were performed because one could argue that mean temperatures of the warmest quarter of the year can be more meaningful and have potentially more impact on such insect species flying during the summer period in a temperate region. Nonetheless, analyses based on mean temperatures of the warmest quarter led to very similar results and the exact same conclusions (results not shown).

**Table 1:** MRDM/LR results and additional parameters derived from CA after having removed the suppressors identified in Table C in S1 File: Pearson’s correlation coefficient *r*, beta weights β, as well as unique, common and total contributions (U, C and T) of environmental distances to the variance in the dependent variable. (*) indicates significant β coefficient values (p-values <0.05 after Benjamini-Hochberg correction).

	*r*	β	U	C	T	*r*	β	U	C	T
**MRDM-CA, response variable:**	**inter-pop. morphometric distance (*mD*)**					**inter-pop. wing size difference (*wsD*)**				
***Melitta leporina***	MRDM R^2^ = 0.086 (p-value = 0.001)					MRDM R^2^ = 0.119 (p-value = 0.001)				
Inter-pop. morphometric distance (*mD*)	-	-	-	-	-	0.121	0.152*	0.022	-0.007	0.015
Inter-pop. wing size difference (*wD*)	0.121	0.157*	0.022	-0.008	0.015	-	-	-	-	-
Geographical distance	0.114	0.093	0.007	0.006	0.013	0.122	0.183*	0.029	-0.014	0.015
Genetic distance (*IID*2)	0.207	0.228*	0.047	-0.004	0.043	-0.237	-0.300*	0.084	-0.028	0.056
Elevation difference	-	-	-	-	-	-	-	-	-	-
Annual mean temperature difference	-0.092	-0.101*	0.009	-0.001	0.008	-0.063	-0.116*	0.012	-0.008	0.004
Annual precipitation difference	-	-	-	-	-	-	-	-	-	-
***Melitta nigricans***	MRDM R^2^ = 0.039 (p-value = 0.003)					MRDM R^2^ = 0.035 (p-value = 0.006)				
Inter-pop. morphometric distance (*mD*)	-	-	-	-	-	0.045	0.040	0.002	0.001	0.002
Inter-pop. wing size difference (*wD*)	0.045	0.037	0.001	0.001	0.002	-	-	-	-	-
Geographical distance	-	-	-	-	-	0.007	0.007	0.000	0.000	0.000
Genetic distance (*IID*2)	0.057	0.057	0.003	0.000	0.003	0.181	0.179*	0.032	0.001	0.033
Elevation difference	0.144	0.113*	0.011	0.009	0.021	-	-	-	-	-
Annual mean temperature difference	0.004	-0.024	0.001	-0.001	0.000	-	-	-	-	-
Annual precipitation difference	0.153	0.119*	0.013	0.011	0.023	-0.016	-0.026	0.001	0.000	0.000
***Melitta tricincta***	MRDM R^2^ = 0.029 (p-value = 0.421)					MRDM R^2^ = 0.103 (p-value = 0.005)				
Inter-pop. morphometric distance (*mD*)	-	-	-	-	-	0.142	0.122	0.015	0.005	0.020
Inter-pop. wing size difference (*wD*)	0.142	0.139	0.018	0.002	0.020	-	-	-	-	-
Geographical distance	-	-	-	-	-	0.192	0.146	0.021	0.017	0.037
Genetic distance (*IID*2)	0.073	0.032	0.001	0.005	0.005	0.253	0.210*	0.042	0.023	0.064
Elevation difference	-	-	-	-	-	-0.092	-0.045	0.002	0.007	0.009
Annual mean temperature difference	-0.047	-0.017	0.000	0.002	0.002	-	-	-	-	-
Annual precipitation difference	-0.081	-0.082	0.006	0.001	0.007	-	-	-	-	-
**LR-CA, response variable:**	**population morphometric diversity (*md*)**					**population mean wing size (*wsM*)**				
***Melitta leporina***	LR R^2^ = 0.177 (p-value = 0.371)					LR R^2^ = 0.154 (p-value = 0.145)				
Pop. morphometric diversity (*md*)	-	-	-	-	-	0.361	0.234	0.121	0.009	0.130
Pop. mean wing size (*wsM*)	0.361	0.476	0.095	0.036	0.130	-	-	-	-	-
Latitude	-	-	-	-	-	-	-	-	-	-
Longitude	-0.251	-0.101	0.005	0.058	0.063	-	-	-	-	-
Nucleotide diversity (*π*)	0.078	0.017	0.000	0.006	0.006	0.183	0.104	0.024	0.009	0.033
Elevation	-	-	-	-	-	-	-	-	-	-
Annual mean temperature	-	-	-	-	-	-	-	-	-	-
Annual precipitation	0.257	0.140	0.010	0.056	0.066	-	-	-	-	-
***Melitta nigricans***	LR R^2^ = 0.130 (p-value = 0.768)					LR R^2^ = 0.188 (p-value = 0.412)				
Pop. morphometric diversity (*md*)	-	-	-	-	-	0.072	0.112	0.017	-0.012	0.005
Pop. mean wing size (*wsM*)	0.072	0.165	0.016	-0.011	0.005	-	-	-	-	-
Latitude	0.209	0.215	0.043	0.001	0.044	-	-	-	-	-
Longitude	0.226	0.232	0.047	0.004	0.051	-0.304	-0.319	0.143	-0.026	0.117
Nucleotide diversity (*π*)	-0.157	-0.106	0.010	0.015	0.025	-0.083	-0.124	0.019	-0.012	0.007
Elevation	-	-	-	-	-	0.102	0.106	0.019	0.021	0.040
Annual mean temperature	-	-	-	-	-	-	-	-	-	-
Annual precipitation	0.017	-0.017	0.000	0.000	0.000	-	-	-	-	-
***Melitta tricincta***	LR R^2^ = 0.260 (p-value = 0.463)					LR R^2^ = 0.279 (p-value = 0.419)				
Pop. morphometric diversity (*md*)	-	-	-	-	-	-0.320	-0.389	0.127	-0.025	0.102
Pop. mean wing size (*wsM*)	-0.320	-0.399	0.152	-0.050	0.102	-	-	-	-	-
Latitude	-	-	-	-	-	-	-	-	-	-
Longitude	-	-	-	-	-	-	-	-	-	-
Nucleotide diversity (*π*)	-0.237	-0.368	0.123	-0.067	0.056	-0.293	-0.376	0.121	-0.035	0.086
Elevation	0.200	0.199	0.034	0.006	0.040	-	-	-	-	-
Annual mean temperature	-	-	-	-	-	0.306	0.189	0.033	0.050	0.083
Annual precipitation	0.006	-0.139	0.018	-0.018	0.000	-	-	-	-	-

CA is a detailed variance-partitioning procedure that can be used to deal with non-independence among spatial predictors [55, 56]. This approach estimates both the “unique” and “common” contributions of predictors to the variance in the response variable. Specifically, unique (U) and common (C) effects respectively represent the amount of variance in the response variable (i.e. in our case the various estimates of inter-individual morphometric variability) that is accounted for by a single predictor and that can be jointly explained by several predictors together [55]. MRDM-CA and LR-CA were performed using R packages “ecodist” [57] and “yhat” [58]. MRDM were based on 1,000 permutations. After the first MRDM/LR-CA analyses, total suppressors were identified and discarded prior to a second series of MRDM/LR-CA analyses. Usually, a total suppressor is a predictor that shares no or little variance with the response variable but that is responsible for artefactual relationships among variables due to the removal of the irrelevant variance in other (suppressed) predictors. Discarding such suppressor variables can potentially purify the relationship between remaining predictors and the response variable [55]. A predictor may be considered a total suppressor when its unique contribution is counterbalanced by its (negative) common contribution or when its regression coefficient and its correlation coefficient are of opposite signs [55].

## Results

### Spatial distribution of wing shape and body size variability

Based on a preliminary visual comparison of interpolation maps displayed in Fig 3, the wing shape and size variability appear unequally distributed in the three species. In other words, such a visual comparison *a priori* does not allow highlighting any convergent pattern for these three sister species. For the shape variability, areas of higher intra-population diversity or inter-population distances do not seem to be shared by the different species (Fig 3A and 3B). These observations are further confirmed by the negative (*M*. *leporina* vs. *M*. *nigricans* and *M*. *nigricans* vs. *M*. *tricincta*) or non-significant (*M*. *leporina* vs. *M*. *tricincta*) inter-specific correlations estimated between interpolation values reported at the different sampling localities (Table B in S1 File). For the wing size (used as a proxy for body size), interpolation graphs clearly highlight an overall higher body size for *M*. *nigricans* (Fig 3C). However, body size does not seem to present any North-South gradient that could have been expected under the assumption of a negative correlation with the temperature (i.e. Bergmann cline).

Furthermore, there is no apparent correlation between inter-population morphometric and genetic distances (Fig 3A vs. Fig 1B), or between intra-population morphometric and genetic diversities (Fig 3B vs. Fig 1C). For instance, while *M*. *leporina* is undoubtedly the species displaying the highest intra-population genetic diversity compared with the two other species (nucleotide diversity; Fig 1C), no such difference is observed for the intra-population morphometric diversity. It is important to note that (i) these first results are only based on preliminary visual comparison of interpolation maps (see below for statistical analyses) and that (ii) similar results are observed when increasing or decreasing the distance weighting parameter *a* (see Figs A and B in S1 File).

### Test of potential predictors of wing shape and size variability

The first series of MRDM/LR-CA’s (Table C in S1 File) has allowed identifying several potential suppressors, i.e. predictors for which Pearson’s correlation coefficient *r* and regression coefficients β are of opposite sign and/or unique contribution is counterbalanced by common contributions [55]. Results of the second series of MRDM-CA and LR-CA’s performed after having discarded potential suppressors are reported in Table 1. Despite low determination coefficients (R^2^ < 0.1), several tested predictors are associated with a significant and positive correlation with inter-population morphometric distance: wing size difference and genetic distance for *M*. *leporina*, as well as elevation and annual precipitation differences for *M*. *nigricans*. Overall, the second series of MRDM-CA and LR-CA results (Table 1) is in agreement with the visual comparison of interpolation graphs: no clear correlation is highlighted between morphometric and genetic inter-individual distances. Indeed, except for *M*. *leporina* (significant coefficient β value and unique contribution of ∼5%; Table 1), genetic distance is not identified as a significant predictor of morphometric distance for the two other species. More generally, as reported in Table 1, these analyses reveal that none of the different predictors show any systematic significant contribution to the variance in inter-population morphometric distances.

For the inter-population wing size difference and also despite similarly low determination coefficient values (R^2^; Table 1), several tested predictors are also associated with a significant and positive correlation: the morphometric and geographic distances as well as the annual mean temperature difference for *M*. *leporina*, the genetic distance for *M*. *nigricans* and *M*. *tricincta*. It is worth noting that in the specific case of *M*. *leporina*, MRDM analysis identifies a significant but negative correlation between inter-population wing size difference and genetic distance. Despite these significant correlations, the same overall result, i.e. the absence of any systematic correlation identified for the three species, is thus also reported for the MRDM-CA analyses of the wing centroid size difference (see Table 1 for further details). While the wing size difference is significantly correlated to the geographical distance for *M*. *leporina* (but with a unique contribution of only about 3%), this is not the case for *M*. *nigricans* and *M*. *tricincta*.

Finally, in the case of the LR-CA’s with the morphometric diversity or the mean wing centroid size as response variable, the overall result is more straightforward: none of the determination coefficient R^2^ values are significant (p-values >> 0.05; Table 1).

## Discussion

### Spatial distribution of morphometric variability: Inter-specific comparison and preliminary explorations

Wing shape variability is unequally distributed within the three sister-species of solitary bees. In particular, hotspots of intra-population morphometric diversity are located in different areas (Fig 3, Table B in S1 File). While locally distinct environmental pressures would have led to convergent spatial heterogeneity, in the case of the three *Melitta* species, wing shape variability is not associated with any detectable or at least obvious common patterns of spatial heterogeneity.

In addition, interpolation graphs of wing shape variability also allow several preliminary visual investigations of the potential impact of tested predictors. For instance, and contrary to what was reported e.g. in [18], the distribution of these morphometric diversity hotspots clearly tends to differ from the distribution of genetic diversity hotspots (Fig 1). Similarly, exploratory visual comparisons of spatial distribution of inter-population genetic and morphometric distances neither allow identifying any clear correspondence between these two measures. Performed for illustrative purposes, such visual analyses are subsequently confirmed by results obtained from testing the different potential predictors.

### Test of potential predictors of wing shape variability

Several tested predictors are identified as potentially important factors of wing shape variability: wing size difference and genetic distance (*M*. *leporina*), as well as elevation and annual precipitation differences (*M*. *nigricans*). Despite these species-specific results, no systematic relationship has been identified between wing shape variability and the predictors tested in this study. In other words, none of the potential factors tested in this study have been identified as a significant predictor for all three species.

It has been demonstrated that geographical distance can be correlated to wing shape variability, e.g., in *Drosophila* [59], Lepidoptera [60] and *Melipona* [61]. Yet, in the present study, we do not identify such correlation on the broad West Palearctic range of the three *Melitta* species. Furthermore, neither a higher genetic diversity seems to imply higher wing shape diversity, nor does a higher level of genetic differentiation suggest a higher level of wing shape differentiation (except for *M*. *leporina* for which there is a significant correlation between morphometric and genetic distances). It has previously been shown that *M*. *leporina* has an overall higher genetic diversity throughout its range, which could be related to an overall higher effective population size allowed by a more abundant floral resource [24]. Yet, this pattern of a higher genetic diversity for *M*. *leporina* is not associated with higher intra-population morphometric diversity. More generally, there is a lack of information about the relationship between morphological and genetic variability at the intra-specific level. Indeed, due to the very low number of studies investigating the relationship between wing morphology and genetic variability at the intra-specific level for insects, the establishment of a general pattern seems hardly possible [62–64]. Among them, [63] have found that higher genetic variability implies higher wing shape variability in populations of butterfly but on the other hand, [64] do not find any concordance. Furthermore, the absence of intra-specific structure of morphometric variability within the three *Melitta* species contrasts with morphometric differentiations highlighted at the inter-specific level, as displayed on the PCoA based on all the different inter-individual distances (Fig C in S1 File). Several insect studies have already combined molecular and geometric morphometrics data at the inter-specific level to assess the taxonomic status of butterflies [65], beetles [66, 67] and cryptic species in bees [68]. Those taxonomic studies have identified congruent patterns between morphological and molecular differentiation between species (e.g. [66]). Globally, this suggests that the “morphometric signal” related to the wing shape variability can be limited to a certain taxonomic level (but see contrasting intra-specific results found by [69] for *Apis mellifera*).

Although the majority of genetic and environmental predictors tested in this study do not seem to explain wing shape variability in the case of the three *Melitta* species, it is clear that wing morphology remains driven by flight performance [70–72]. Those flight performances could be the main driver of wing morphology as it is under tight genetic control of the complex interplay of multiple loci [73–75], which could be a reason for the low impact of the tested predictors. Three additional hypotheses could also be proposed to explain the absence of systematic intra-specific wing shape/size variability predictors. Firstly, several non-investigated factors could potentially explain this variability, like, for instance, the fragmentation of the habitat and the type of landscapes [13, 76]. Secondly, wing shape variability could also be shaped by sexual selection as in some other insect species (e.g. wing coloration in *Calopteryx* species [14]). Finally, as the wing shape variability is unequally distributed and as none of the investigated predictors were systematically correlated with it, the distribution of the intra-specific variability could simply be random. This last assumption implies that this intra-specific variability is not affected by strong local selection pressures.

### Test of potential predictors of wing size variability

Similarly, no common pattern of wing centroid size (i.e. proxy for the total body size) variability is found in this study. If applied to insects, Bergmann’s rule (initially formulated for endotherm vertebrates) would predict that individuals from warmer climates would have smaller body size than individuals from colder climates [27]. Our results corroborate previous studies where no such pattern has been found for several insect groups (e.g. Lepidoptera [77]; see also [28] for a review). In beetle species, Bergmann clines, converse Bergmann clines or even no clines at all can appear [78, 79]. In addition, even when individuals are larger at higher latitudes, body size is not systematically correlated with a gradient of temperature, suggesting that other environmental factors (e.g. precipitation, seasonality, life cycle length) can be responsible of the Bergmann cline [79, 80]. Body size of American bees can also show the three types of clines depending on families [81]. Since it has been reported in previous studies that many species do not follow such “Bergmann clines”, our results further suggest that this rule does not apply systematically for bee species.

While the wing centroid size seems to represent a reasonable proxy for body size [13], its relevance as such a proxy could be discussed for at least two reasons. Firstly, according to Allen’s rule [82], animals tend to have relatively shorter limbs such as wings in colder climates. Our results have not highlighted such a trend for the three *Melitta* species, but the opposite situation could have compromised the use of the wing centroid size as a proxy for body size. Secondly, the endothermy that has been demonstrated for several bee species during flight could have a potential impact on wing size selection if such selection occurs for flight performance. As a consequence, while useful, results based on this proxy have to be interpreted with caution. Furthermore, bee species can exhibit very different morphologies or life history traits (e.g. phenology, sociability and nesting behaviour). Consequently, it could be relevant to re-assess this relationship between body size and climate according to the different taxonomic levels (e.g. genus) or to different life history traits. This would allow investigating the correlation between body size and temperature in a genus-based or trait-based context. Indeed, even if the body size of these *Melitta* species does not vary with respect to temperature, some bee taxa like bumblebees could be more impacted, mainly because of their endothermy. Yet, even if bumblebees in the United Kingdom are larger in cooler climates both at the intra- and interspecific levels [32], tropical bumblebees tend to be the largest of all [32], despite considerable body size variations among species.

## Supporting information

S1 File Supplementary Figures A, B and C, and Supplementary Tables A, B and C. Figure A: interpolation graphs generated with a distance weighting parameter *a* = 1. Figure B: interpolation graphs generated with a distance weighting parameter *a* = 10. Figure C: PCoA (principal component analysis) performed on the overall matrix of estimated morphological distances. Table A: sampling localities of *Melitta leporina*, *nigricans* and *tricincta*. Table B: for each pair of species, correlation statistics between intra-population morphometric diversity values. Table C: MRDM/LR results and additional parameters derived from CA for different response variables.
(PDF)

S2 File Morphometric data. (ZIP)
