# Supplementary material for: Distribution and predictors of wing shape and size variability in three sister species of solitary bees
Source: PLoS One. 2017 Mar 8;12(3):e0173109. doi: 10.1371/journal.pone.0173109 (PMC5342212; doi:10.1371/journal.pone.0173109)
Supplement: S1 File — Figure A: interpolation graphs generated with a distance weighting parameter a = 1. Figure B: interpolation graphs generated with a distance weighting parameter a = 10. Figure C: PCoA (principal component analysis) performed on the overall matrix of estimated morphological distances. Table A: sampling localities of Melitta leporina, nigricans and tricincta. Table B: for each pair of species, correlation statistics between intra-population morphometric diversity values. Table C: MRDM/LR results and additional parameters derived from CA for different response variables. (PDF) [file pone.0173109.s001.pdf]

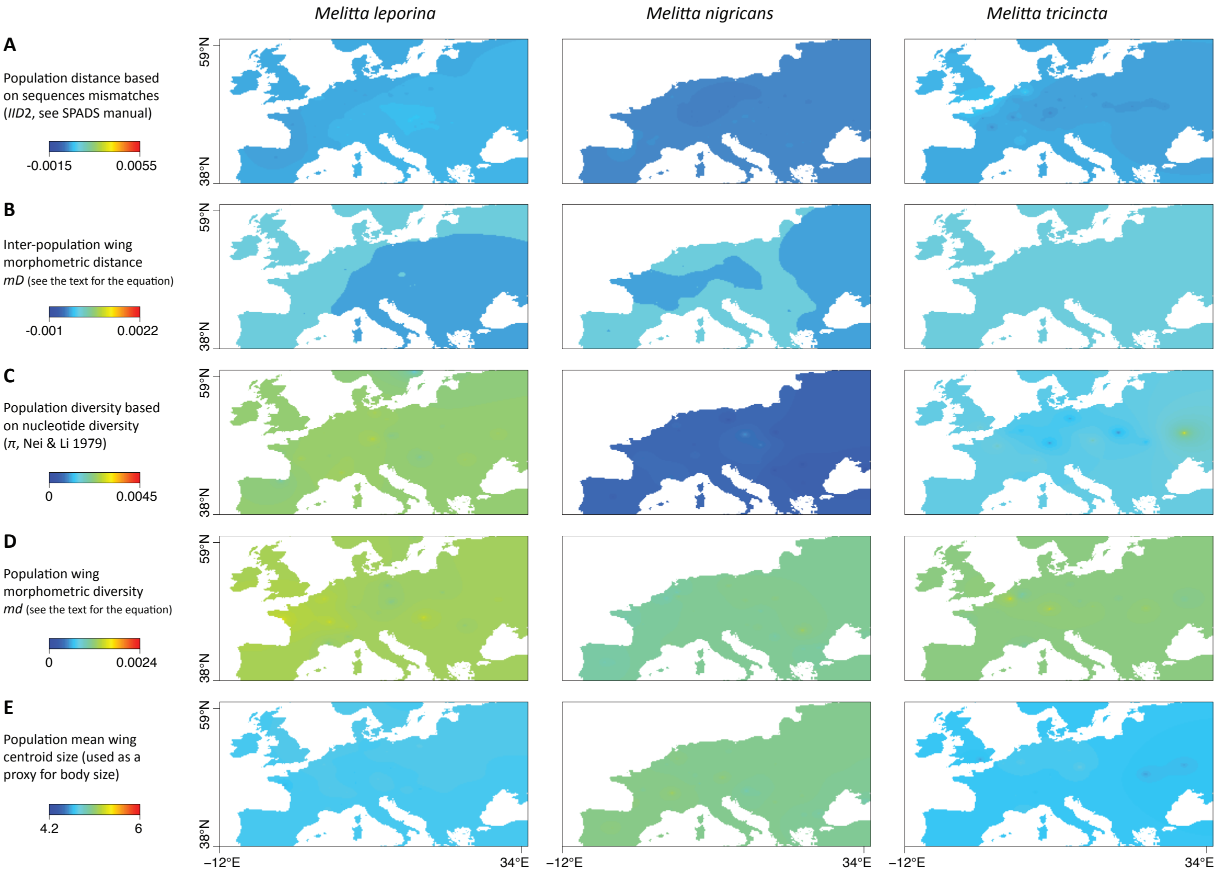

Fig A. Interpolation graphs generated with a distance weighting parameter *α* = 1: population genetic distance based on DNA sequence mismatches (*IID2*; **A**), Inter-population morphometric distance (*mD*; **B**), population nucleotide diversity (*π*; **C**), population morphometric diversity (*md*; **D**) and population mean wing centroid size (*wsM*; used as a proxy for body size; **E**).

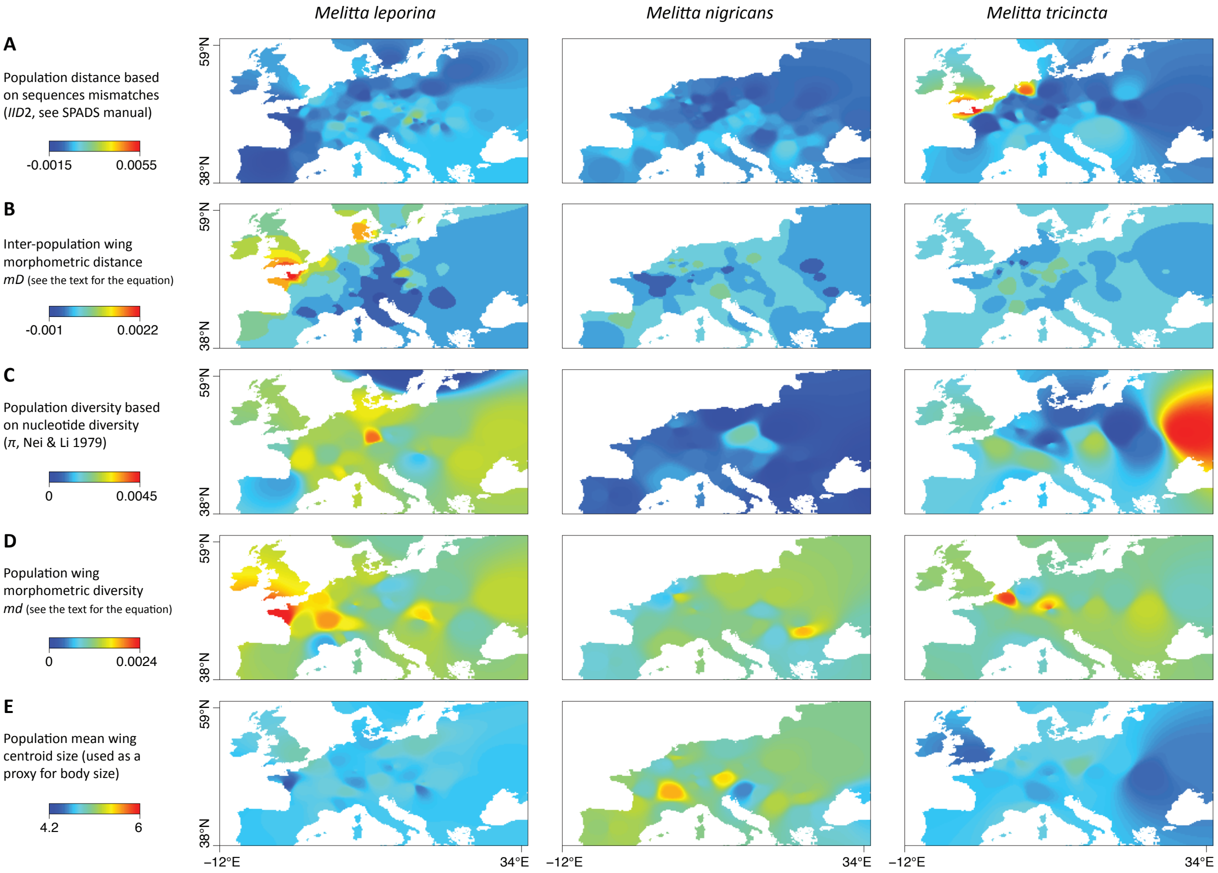

Fig B. Interpolation graphs generated with a distance weighting parameter *α* = 10: population genetic distance based on DNA sequence mismatches (*IID2*; **A**), Inter-population morphometric distance (*mD*; **B**), population nucleotide diversity (*π*; **C**), population morphometric diversity (*md*; **D**) and population mean wing centroid size (*wsM*; used as a proxy for body size; **E**).

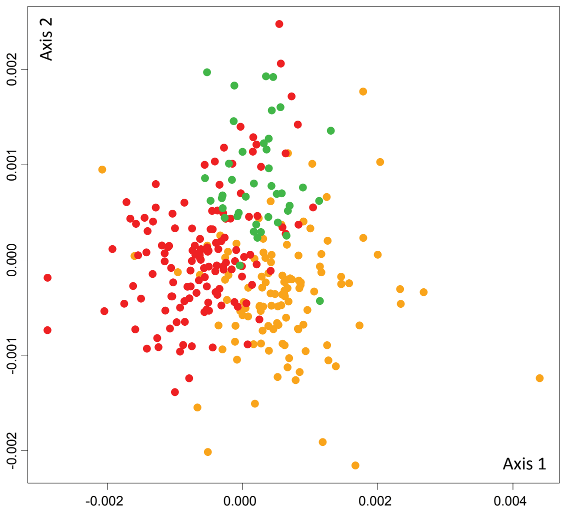

Fig C. PCoA (principal component analysis) performed on the overall matrix of estimated morphological distances. The dots are coloured according to the three species: *M. leporina* (orange), *M. nigricans* (red) and *M. tricincta* (green).

Table A. Sampling localities of *Melitta leporina*, *nigricans* and *tricincta* (see Table S2 in Dellicour *et al*. [2015] for more details about the genetic data available for each sampled population). "*n* (COI)" refers to the number of sampled individuals sequenced at the mitochondrial locus COI and "*n* (morpho)" refers to the number of sampled individuals for which morphometric data were generated.

Population Locality	Geographical coordinates	*n* (COI)	*n* (morph)	Population Locality	Geographical coordinates	*n* (COI)	*n* (morph)	Population Locality	Geographical coordinates	*n* (COI)	*n* (morph)
Melitta leporina				***Melitta nigricans***				***Melitta tricincta***			
Mons (Belgium)	50.46°N, 3.96°E	3	3	Erbisoeul (Belgium)	50.52°N, 3.92°E	5	5	Boussoit (Belgium)	50.47°N, 4.08°E	2	3
Beroun (Czech Republic)	49.97°N, 14.07°E	4	3	Beli Osam (Bulgaria)	42.86°N, 24.67°E	7	9	Boří (Czech Republic)	49.28°N, 16.63°E	4	9
Chvalské Skály (Czech Republic)	50.11°N, 14.60°E	1	1	Smyadovo (Bulgaria)	43.03°N, 27.05°E	7	11	Klánovicky (Czech Republic)	50.08°N, 14.67°E	3	6
Prague (Czech Republic)	50.11°N, 14.42°E	5	4	Kolin (Czech Republic)	50.05°N, 15.21°E	1	5	Surrey (England)	51.25°N, 0.47°W	3	8
Pegwell Bay (England)	51.30°N, 1.37°E	2	1	Na Plachtě (Czech Republic)	50.18°N, 15.85°E	3	12	Essonne (France)	48.45°N, 2.15°E	11	8
Arles (France)	43.64°N, 4.58°E	3	6	Arles (France)	43.64°N, 4.58°E	7	8	Gonfaron (France)	43.32°N, 6.35°E	8	15
Barcelonnette (France)	44.38°N, 6.61°E	3	3	Cap Feret (France)	44.78°N, 1.15°W	2	1	Lille (France)	50.65°N, 3.04°E	1	1
Batz sur Mer (France)	47.28°N, 2.48°W	2	2	Les Grandes Bruyères (France)	46.15°N, 4.74°E	2	2	Rillieux-la-Pape (France)	45.80°N, 4.90°E	3	1
Donges (France)	47.32°N, 2.07°W	1	2	Poitiers (France)	46.57°N, 0.34°E	4	8	Saint-Lyphard (France)	47.40°N, 2.31°W	7	8
Poitiers (France)	46.55°N, 0.41°E	10	13	Achern (Germany)	48.64°N, 8.06°E	5	5	Berlin (Germany)	52.60°N, 13.16°E	7	10
Puligny-Montrachet (France)	46.95°N, 4.77°E	2	3	Berlin (Germany)	52.45°N, 13.33°E	3	2	Giengen (Germany)	48.63°N, 10.25°E	4	3
Berlin (Germany)	52.45°N, 13.56°E	2	2	Gerzen (Germany)	48.51°N, 12.43°E	2	1	Onatsfeld (Germany)	48.87°N, 10.08°E	3	5
Dorfen (Germany)	48.28°N, 12.15°E	2	2	Reichenschwad (Germany)	49.51°N, 11.38°E	2	2	Maarland (Holland)	50.79°N, 5.70°E	3	9
Heidelberg (Germany)	49.40°N, 8.68°E	2	4	Webenheim (Germany)	49.25°N, 7.28°E	1	2	Merum (Holland)	51.17°N, 5.94°E	2	3
Klinken (Germany)	53.49°N, 11.67°E	3	3	Winkel (Germany)	48.83°N, 8.31°E	4	7	Zakrzów (Poland)	50.48°N, 20.53°E	8	6
Reilingen (Germany)	49.29°N, 8.58°E	5	4	Eindhoven (Holland)	51.42°N, 5.48°E	4	4	Brigerbad (Switzerland)	46.30°N, 7.92°E	4	16
Biesland (Holland)	50.83°N, 5.69°E	2	3	Gronsveld (Holland)	50.81°N, 5.73°E	4	7	Kiev (Ukraine)	50.40°N, 30.54°E	8	4
Budapest (Hungary)	47.57°N, 19.13°E	2	2	Neder-Betuwe (Holland)	51.94°N, 5.62°E	1	1	Tiaziv (Ukraine)	49.02°N, 24.70°E	8	6
Rakamaz (Hungary)	48.13°N, 21.46°E	5	5	Szigetvár (Hungary)	46.05°N, 17.86°E	10	14				
Simontornya (Hungary)	46.75°N, 18.53°E	3	4	Pinczow (Poland)	50.50°N, 20.47°E	7	4				
Szentendrei (Hungary)	47.66°N, 19.07°E	3	3	Cârţişoara (Romania)	45.68°N, 24.58°E	2	2				
Zalaszabar (Hungary)	46.64°N, 17.11°E	1	1	Târnăveni (Romania)	46.44°N, 24.25°E	12	22				
Piemont (Italy)	44.69°N, 7.60°E	8	8	Belgrade (Serbia)	44.78°N, 20.37°E	7	4				
Dorposz Szlachecki (Poland)	53.29°N, 18.43°E	1	1	Rajčany (Slovakia)	48.61°N, 18.23°E	5	9				
Putawy (Poland)	51.42°N, 21.97°E	8	9	Savci (Slovenia)	46.48°N, 16.06°E	5	7				
Zetea (Romania)	46.39°N, 25.37°E	8	8	Aguilar de Campoo (Spain)	40.79°N, 4.29°W	8	12				
Sekule (Slovakia)	48.60°N, 17.00°E	1	1	Alba de Tormes (Spain)	40.82°N, 5.52°W	8	10				
Amorebieta (Spain)	43.18°N, 2.63°W	10	10	Aesch (Switzerland)	47.26°N, 8.23°E	5	8				
Vegas de Matute (Spain)	40.79°N, 4.18°W	1	2	Frauenfeld (Switzerland)	47.57°N, 8.92°E	1	3				
Uppsala (Sweden)	59.69°N, 17.68°E	5	2	Crimea (Ukraine)	44.87°N, 34.22°E	6	5				
Diessenhofen (Switzerland)	47.68°N, 8.76°E	2	1	Kiev (Ukraine)	50.33°N, 30.47°E	3	1				
Sion (Switzerland)	46.21°N, 7.39°E	2	2	Camoël (France)	47.48°N, 2.40°W	3	9				
Kiev (Ukraine)	50.33°N, 30.47°E	8	8								

Table B. For each pair of species, correlation statistics between intra-population morphometric diversity values. As these species were not sampled at the exact same locations, these correlation statistics were based on values extracted from interpolation graphs at the same locations. To avoid as much extrapolation as possible, we only considered morphometric diversity values reported for sampling localities. In practice, md values were extracted from the corresponding interpolation maps (Fig 3B, maps generated with a distance weighting parameter a = 5) with the combined set of sampling geographic coordinates of all the three species (total of 83 localities). *r*, β and R^2 respectively refer to Pearson's correlation coefficient, linear regression beta weight and determination coefficient (reported with their p-value).

		Linear regression		
	r	**β**	**R^2**	**p-value**
M. leporina vs. *M. nigricans*	-0.567	-1.010	0.322	< 0.001
M. nigricans vs. *M. tricincta*	-0.357	-0.240	0.127	0.001
M. leporina vs. M. tricincta	0.180	0.129	0.012	0.330

Table C. MRDM/LR results and additional parameters derived from CA for different response variables: Pearson's correlation coefficient *r*, beta weights β, as well as unique, common and total contributions (U, C and T) of environmental distances to the variance in the dependent variable. (*) indicates significant β coefficient values (p-values <0.05 after Benjamini-Hochberg correction).

	r	β	U	C	T	*r*	β	U	C	T
**MRDM-CA, response variable:**	**inter-pop. morphometric distance (*mD*)**					**inter-pop. wing size difference (*wsD*)**				
Melitta leporina	MRDM R^2 = 0.089 (p-value = 0.001)					MRDM R^2 = 0.133 (p-value = 0.001)				
Inter-pop. morphometric distance (*mD*)	-	-	-	-	-	0.121	0.153*	0.022	-0.007	0.015
Inter-pop. wing size difference (*wsD*)	0.121	0.160*	0.023	-0.008	0.015	-	-	-	-	-
Geographical distance	0.114	0.089	0.006	0.007	0.013	0.122	0.214*	0.038	-0.023	0.015
Genetic distance (*IID*2)	0.207	0.234*	0.048	-0.005	0.043	-0.237	-0.318*	0.093	-0.036	0.056
Elevation difference	-0.022	-0.038	0.001	-0.001	0.001	-0.018	-0.031	0.001	-0.001	0.000
Annual mean temperature difference	-0.092	-0.107*	0.010	-0.002	0.008	-0.063	-0.106*	0.010	-0.006	0.004
Annual precipitation difference	0.004	0.041	0.002	-0.002	0.000	-0.048	-0.116*	0.012	-0.010	0.002
Melitta nigricans	MRDM R^2 = 0.056 (p-value = 0.001)					MRDM R^2 = 0.039 (p-value = 0.008)				
Inter-pop. morphometric distance (*mD*)	-	-	-	-	-	0.045	0.040	0.002	0.001	0.002
Inter-pop. wing size difference (*wsD*)	0.045	0.039	0.002	0.001	0.002	-	-	-	-	-
Geographical distance	-0.071	-0.136*	0.016	-0.011	0.005	0.007	0.021	0.000	0.000	0.000
Genetic distance (*IID*2)	0.057	0.058	0.003	0.000	0.003	0.181	0.194*	0.036	-0.003	0.033
Elevation difference	0.144	0.142*	0.017	0.003	0.021	-0.010	0.002	0.000	0.000	0.000
Annual mean temperature difference	0.004	0.001	0.000	0.000	0.000	-0.025	-0.072	0.005	-0.004	0.001
Annual precipitation difference	0.153	0.134*	0.016	0.007	0.023	-0.016	-0.028	0.001	0.000	0.000
Melitta tricincta	MRDM R^2 = 0.030 (p-value = 0.678)					MRDM R^2 = 0.126 (p-value = 0.010)				
Inter-pop. morphometric distance (*mD*)	-	-	-	-	-	0.142	0.130	0.017	0.003	0.020
Inter-pop. wing size difference (*wsD*)	0.142	0.144	0.019	0.002	0.020	-	-	-	-	-
Geographical distance	0.013	-0.016	0.000	0.000	0.000	0.192	0.157	0.022	0.015	0.037
Genetic distance (*IID*2)	0.073	0.036	0.001	0.004	0.005	0.253	0.213	0.043	0.021	0.064
Elevation difference	-0.047	0.027	0.001	0.002	0.002	-0.092	-0.110	0.008	0.001	0.009
Annual mean temperature difference	-0.047	-0.019	0.000	0.002	0.002	-0.026	-0.069	0.004	-0.003	0.001
Annual precipitation difference	-0.081	-0.095	0.006	0.000	0.007	0.065	0.174	0.021	-0.017	0.004
**LR-CA, response variable:**	**population morphometric diversity (*md*)**					**population mean wing size (*wsM*)**				
Melitta leporina	LR R^2 = 0.184 (p-value = 0.765)					LR R^2 = 0.205 (p-value = 699)				
Pop. morphometric diversity (*md*)	-	-	-	-	-	0.361	0.204	0.084	0.046	0.130
Pop. mean wing size (*wsM*)	0.361	0.469	0.087	0.044	0.130	-	-	-	-	-
Latitude	-0.091	0.030	0.000	0.008	0.008	-0.026	0.401	0.037	-0.030	0.007
Longitude	-0.251	-0.090	0.002	0.061	0.063	-0.026	0.133	0.010	0.021	0.031
Nucleotide diversity (π)	0.078	0.052	0.002	0.004	0.006	0.183	0.160	0.043	-0.010	0.033
Elevation	0.005	-0.108	0.001	-0.001	0.000	0.207	0.293	0.023	-0.023	0.000
Annual mean temperature	0.136	-0.042	0.000	0.018	0.019	-0.053	0.421	0.040	-0.017	0.023
Annual precipitation	0.257	0.209	0.014	0.052	0.066	-0.206	0.119	0.010	0.015	0.025
Melitta nigricans	LR R^2 = 0.178 (p-value = 0.845)					LR R^2 = 0.230 (p-value = 0.717)				
Pop. morphometric diversity (*md*)	-	-	-	-	-	0.072	0.065	0.005	0.000	0.005
Pop. mean wing size (*wsM*)	0.072	0.099	0.005	0.000	0.005	-	-	-	-	-
Latitude	0.209	0.561	0.017	0.026	0.044	-0.008	0.314	0.008	-0.007	0.001
Longitude	0.226	0.342	0.020	0.031	0.051	-0.304	-0.248	0.015	0.102	0.117
Nucleotide diversity (π)	-0.157	-0.065	0.003	0.021	0.025	-0.083	-0.111	0.014	-0.007	0.007
Elevation	0.044	0.414	0.014	-0.012	0.002	0.102	0.278	0.009	0.031	0.040
Annual mean temperature	-0.364	0.189	0.002	0.131	0.133	0.051	0.127	0.001	-0.001	0.000
Annual precipitation	0.017	0.054	0.002	-0.001	0.000	0.065	-0.117	0.011	0.006	0.017
Melitta tricincta	LR R^2 = 0.330 (p-value = 0.768)					LR R^2 = 0.387 (p-value = 0.660)				
Pop. morphometric diversity (*md*)	-	-	-	-	-	-0.320	-0.466	0.173	-0.071	0.102
Pop. mean wing size (*wsM*)	-0.320	-0.472	0.189	-0.087	0.102	-	-	-	-	-
Latitude	0.079	0.921	0.052	-0.045	0.006	0.005	1.050	0.064	-0.064	0.000
Longitude	-0.014	0.187	0.008	-0.008	0.000	-0.222	0.274	0.016	0.018	0.033
Nucleotide diversity (π)	-0.237	-0.286	0.068	-0.012	0.056	-0.293	-0.292	0.066	0.020	0.086
Elevation	0.200	1.117	0.074	-0.034	0.040	-0.206	1.207	0.082	-0.055	0.028
Annual mean temperature	-0.169	1.138	0.044	-0.016	0.028	0.306	1.536	0.079	0.004	0.083
Annual precipitation	0.006	0.090	0.002	-0.002	0.000	-0.155	0.114	0.003	0.018	0.022
